# Supplementary material for: Grafted Sertoli Cells Exert Immunomodulatory Non-Immunosuppressive Effects in Preclinical Models of Infection and Cancer
Source: Cells. 2024 Mar 19;13(6):544. doi: 10.3390/cells13060544 (PMC10969358; doi:10.3390/cells13060544)
Supplement: Supplementary file 1 [file cells-13-00544-s001.zip › cells-2882273-suppl.pdf]

SUPPLEMENTARY MATERIAL

Table S1. Primers used in real-time PCR analyses.

Gene	Forward primer 5′-3′	Reverse primer 5′-3′
Actb	ATTACTGCTCTGGCTCCTA	ATCTGCTGGAAGGTGGAC
Ccr2	AGAGAGCTGCAGCAAAAGG	GGAAAGAGGCAGTTGCAAAG
Cxcl1	CCGCTCGCTTCTCTGTGC	CTCTGGATGTTCTTGAGGTGAATC
Cyp1a1	ACAGTGATTGGCAGAGATCG	GAAGGGGACGAAGGATGAAT
Cyp1b1	TTCTCCAGCTTTTTGCCTGT	TAATGAAGCCGTCCTTGTCC
Gapdh	*GCCTTCCGTGTTCCTACCC*	*CAGTGGGCCCTCAGATGC*
Ido1	CCCACACTGAGCACGGACGG	GCCCTTGTCGCAGTCCCCAC
Il1b	TGA CGG ACC CCA AAA GAT GAA GG	CCA CGG GAA AGA CAC AGG TAG C
Il1ra	TTGTGCCAAGTCTGGAGATG	CAGCTGACTCAAAGCTGGTG
Il6	CCGGAGAGGAGACTTCACAG	TCCACGATTTCCCAGAGAAC
Il10	GAGAAGCATGGCCCAGAAATCAAG	ATCACTCTTCACCTGCTCCACTGC
Il17a	GACTACCTCAACCGTTCCAC	CCTCCGCATTGACACAGC
Il22	CTGCCTGCTTCTCATTGCCCTGTG	GATGTACGGCTGCTGGAAGTTGG
Mmp2	AACGGTCGGGAATACAGCAG	ATGGGTTGGATCTTCATGGGG
Mmp9	TAGCACAACAGCTGACTACG	ATCCTGGTCATAGTTGGCTG
S100a8	TCGTGACAATGCCGTCTGAACTG	TGCTACTCCTTGTGGCTGTCTTTG
S100a9	CGCAGCATAACCACCATCATC	GCCATCAGCATCATACACTCC
Snai1	CTTGTGTCTGCACGACCTGT	CATCCGAGTGGGTTTGGAGG
Twist1	CTGCCCTCGGACAAGCTGAG	CTAGTGGGACGCGGACATGG
Vegfa	TATTCAGCGGACTCACCAGC	AACCAACCTCCTCAAACCGT
Vegfb	TGACGATGGCCTGGAATGTG	GAGGATCCTGGGGCTGTCT
Vegfc	GCTGATGTCTGTCCTGTACCC	AGAAGGTGTTTGTGGCTGCT
Vegfr2	TCCACATGGGCGAATCACTC	GCAATTCTGTCACCCAGGGA
Zeb1	GGAGAGGTGACTGGTTGTGG	GCCACATCAGCAATAGCAGC

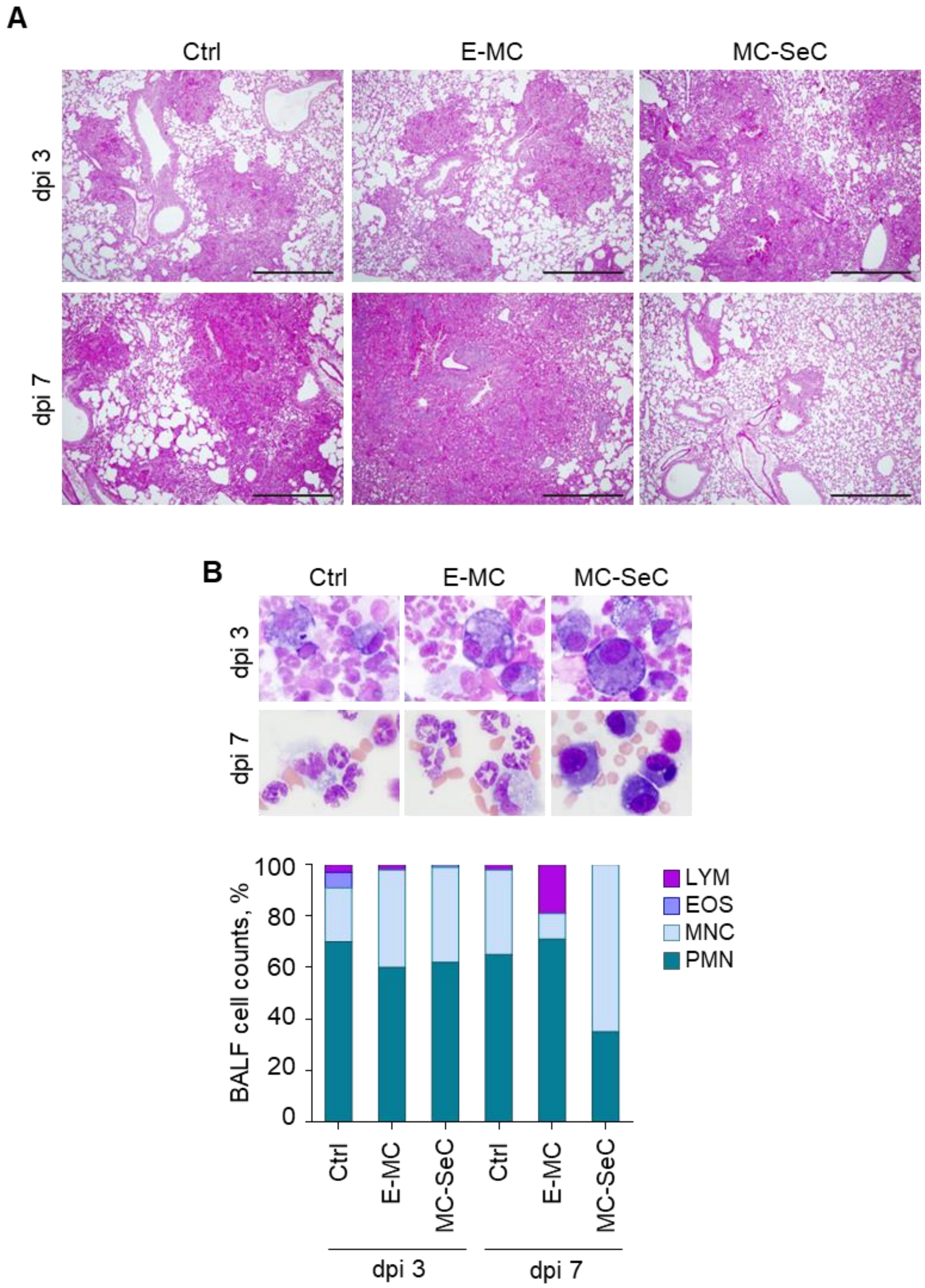

Figure S1. Grafted SeC protect from *A. fumigatus* intratracheal infection. (A,B) Control mice (Ctrl) and mice injected i.p. with MC-SeC (1.0x10^6 SeC/g body weight) or equivalent amount of E-MC were infected intratracheally with *A. fumigatus* conidia. Periodic acid-Schiff staining of lungs (A), and BALF morphometry analysis (B) were performed at 3 and 7 days post-infection (dpi). Reported are BALF differential cell counts. Scale bars (A), 1 mm; original magnification (B), 100X.

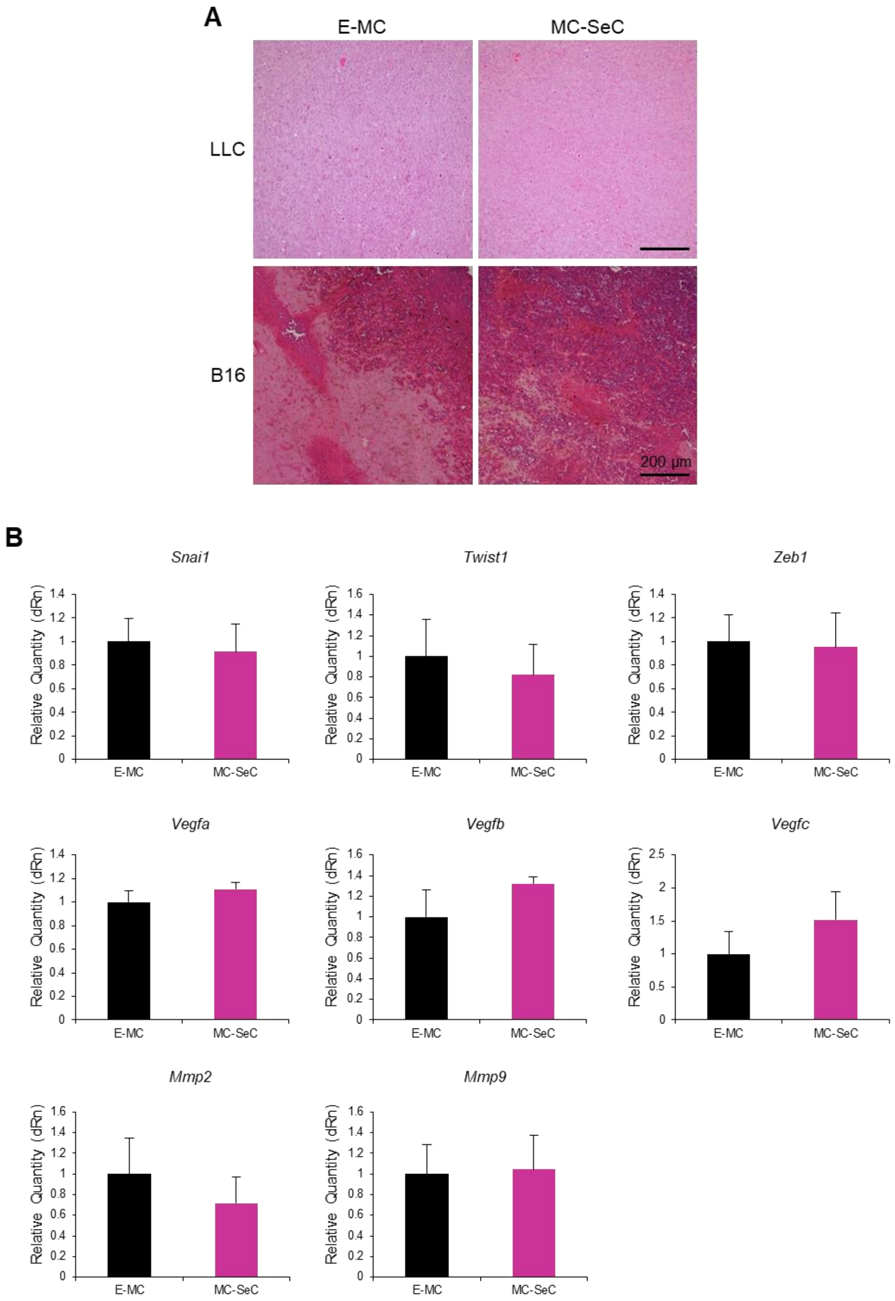

Figure S2. Grafted SeC do not affect primary tumor histology. (A) Primary LLC and B16 tumor masses developed in mice injected i.p. with MC-SeC (1.0x10^6 SeC/g body weight) or equivalent amount of E-MC were histologically evaluated after hematoxylin-eosin staining. Shown are representative images. Scale bars, 200 µm. **(B)** LLC tumor masses were evaluated for the expression of epithelial-mesenchymal transition markers (*Snai1*, *Twist1*, and *Zeb1*), matrix metalloproteases (*Mmp2* and *Mmp9*), and angiogenesis markers (*Vegfa*, *Vegfb*, and *Vegfc*) by real-time PCR.
